# False positive endobronchial ultrasound-guided real-time transbronchial needle aspiration secondary to bronchial carcinoma in situ at the point of puncture: a case report

**DOI:** 10.1186/1749-8090-7-74

**Published:** 2012-08-14

**Authors:** José Sanz-Santos, Felipe Andreo, Pere Serra, María Llatjós, Eva Castellà, Julio Astudillo, Eduard Monsó, Juan Ruiz-Manzano

**Affiliations:** 1 Pulmonology Department, Hospital Universitari Germans Trias i Pujol, Carretera de Canyet S/N, 08916, Badalona, Barcelona, Spain; 2 Medicine Department, School of Medicine, Universitat Autònoma de Barcelona, Bellaterra, Barcelona, Spain; 3 Ciber de Enfermedades Respiratorias (CiBERES), Bunyola, Balearic Islands, Spain; 4 Pathology Department, Hospital Universitari Germans Trias i Pujol, Carretera de Canyet S/N, 08916, Badalona, Barcelona, Spain; 5 Thoracic Surgery Department, Hospital Universitari Germans Trias i Pujol, Carretera de Canyet S/N, 08916, Badalona, Barcelona, Spain; 6 Pulmonology Department, Hospital Parc Taulí, Sabadell, Barcelona, Spain

**Keywords:** Lung Cancer, Endobronchial Ultrasound, Staging, False Positive, Carcinoma in situ

## Abstract

Since the development of endobronchial ultrasound-guided real-time needle aspiration (EBUS-rt-TBNA) no false positive (FP) cases have been described. We present the first FP case for EBUS-rt-TBNA secondary to a carcinoma in situ (CIS) in the bronchial point of puncture. A 66-years-old male was referred to our Institution because of a mass in left lower lobe. The bronchoscopy did not show any endobronchial lesion. The cytology of the washing confirmed an unspecified non-small cell lung cancer. An EBUS-rt-TBNA for staging was carried out. No mediastinal nodes over 5 mm length were found but one single left hilar node at station 11 L was sampled. The cytology of the TBNA showed lymphocytes and neoplastic squamous cells. The patient underwent thoracotomy. On the surgical specimen no metastasis on any of the nodes resected were detected but a CIS on the bronchial resection margin was described. A bronchial biopsy confirmed CIS on the bronchial stump. The reported case depicts an unusual situation, we consider EBUS-rt-TBNA an accurate technique if minimal requirements are met

## Background

Endobronchial ultrasound-guided real-time transbronchial needle aspiration (EBUS-rt-TBNA) is a relatively novel technique that has proven useful in lung cancer diagnosis and staging. EBUS-rt-TBNA can be performed under conscious sedation in an outpatient setting. Several studies have demonstrated that EBUS-rt-TBNA is an accurate procedure alternative to surgical staging, with fewer complications and similar figures for sensitivity and specificity. While an average false-negative rate of 10 % represents a handicap for this procedure, no false-positive (FP) cases have been described. We present the first FP case of EBUS-rt-TBNA.

## Case report

**Figure 1:**
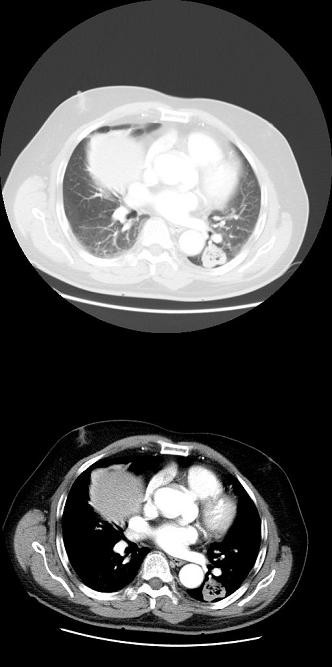
Thoracic CT-scan: Peripheral mass in left lower lobe with central cavitation.

**Figure 2:**
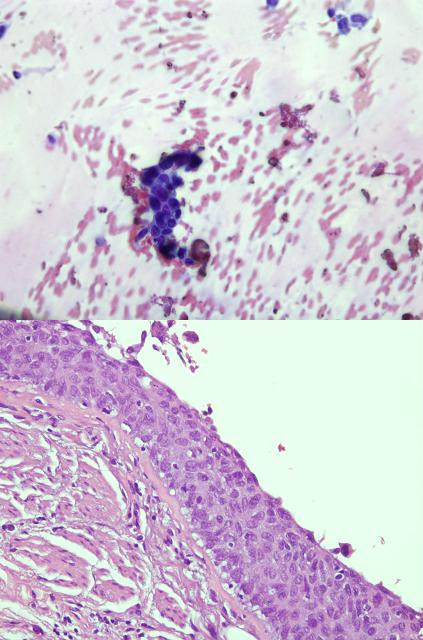
a: Transbronchial lymph node aspiration specimen: cluster of neoplastic squamous cells (no lymphocytes in this field) (Papanicolau stain, x400) b: Surgical specimen: carcinoma in situ (CIS) on the bronchial resection margin (Haematoxylin&Eosin stain, x400).

A 66-year-old male, with a 40 pack-year smoking history, consulted his general practitioner because of persistent cough lasting for 3 months. A chest x-ray was performed and a mass on left lower lobe (LLL) was detected. The patient was then referred to our Institution. The thoracic CT-scan confirmed the presence of a peripheral mass on LLL (4x3 cm), without evidence of nodal enlargement (Figure 1). A white light bronchoscopy was performed and no endobronchial lesions were detected. The bronchial washing cytology was positive for unspecified non-small cell lung cancer. An EBUS-rt-TBNA for staging was performed. There were no nodes over 5 mm in short-axis diameter on mediastinal stations but one left hilar node, at 11 L station, measuring 12x9 mm was detected and sampled. The cytological examination of the smear showed the presence of lymphocytes and a few groups of neoplastic squamous cells (Figure 2a). The patient was diagnosed with squamous-cell carcinoma (SCC) stage IIa cT2aN1M0 and underwent left lower lobe lobectomy. The surgical specimen consisted of a peripheral mass in LLL measuring 4x3 cm consistent with SCC, carcinoma in situ (CIS) on the bronchial resection margin (Figure 2b) without nodal involvement of any of the 9 nodes resected. Several cuts of the hilar nodes were carried out but no neoplastic cells were detected. The postsurgical staging was pT2aN0M0 with CIS on the bronchial resection margin. A few weeks later a bronchoscopy with autofluorescence was performed. An area of low autofluorescence extending from the lobectomy stump to the main left bronchus was detected; the bronchial biopsy confirmed the CIS. The CIS was treated twice with endobronchial argon plasma coagulation. Due to local recurrence, the patient finally underwent pneumonectomy.

## Discussion

Since the development of transbronchial needle aspiration (TBNA) for flexible bronchoscopy for lung cancer staging in the eighties, false-positives (FP) have been rarely reported. A case is considered as FP when tumor cells are identified on transbronchial lymph node aspiration but tumor metastases are not found in nodes obtained by thoracotomy or mediastinoscopy. Probably the exceptionality of FP in TBNA sampling is an accurate estimation but not all the studies confirmed positive TBNA results with further invasive procedures. When the technique was first described [1] it was recommended to perform the TBNA prior to any manipulation in order to minimize the risk of contamination of the aspiration specimen and then avoid potential false-positive results.

Cropp [2] and cols were the first investigators to describe a false positive in a TBNA sampling. These authors postulated that tumor cells exfoliated from bronchogenic carcinoma could be located on the mucosa surface and trapped as the needle penetrated the tracheal wall and could therefore be collected during aspiration. Other authors described a FP case probably secondary to tumor sampling instead of lymph node [3]. For this reason, special care was recommended when an aspiration harboured neoplastic cells but no lymphocytes, especially in patients with minor radiological suspicion. Other cases of FP TBNA were attributed to needle contamination but overall TBNA has been considered highly reliable and FP have been considered clinically non-significant [4].

With the emergence of echo-probes for radial EBUS the accuracy of TBNA procedures increased and only one case of FP was registered in a series of Okamoto et al. [5] Since the introduction of echobronchoscopes, that provide real time assistance to TBNA no false-positive transbronchial lymph node aspirates have been described in any series, and consequently a specificity of 100 % has been conferred to this technique. Although, as previously, only a few studies performed with EBUS-rt-TBNA confirmed positive results with mediastinoscopy or thoracotomy.

EBUS allows real time guidance of the puncture avoiding incidental sampling of masses. The needle used for EBUS-rt-TBNA incorporates a stylet in the inner channel that prevents contamination. The only potential scenario for FP results of EBUS-rt-TBNA is the use of a single needle to sample more than one node after a positive result.

To our knowledge, the present case is the first FP documented for EBUS-rt-TBNA and is the first attributed to needle contamination by neoplastic cells from a bronchial CIS on the puncture point. As a mechanism similar to that suggested by Cropp and cols, probably malignant squamous cells from the CIS were introduced during the puncture and then aspirated. During the procedure the syringe suction was always attached and released just when the needle was inside the lymph node. Consequently, we do not consider incidental suction of the bronchial wall as a possible explanation for this case. Fortunately, in our patient the FP had no consequences on the staging that could change the choice of treatment. CIS are frequent in smoking patients with lung neoplasm, may be multicentric and difficult to identify with white light bronchoscopy [6].

## Conclusions

The present case represents a fortuitous coincidence that might be considered when obtained results are contradictory in patients with squamous lung carcinoma. EBUS-rt-TBNA is a highly specific technique with a very low risk of contamination if minimal requirements are met.

## Consent

Written informed consent was obtained from the patient for publication of this Case report and any accompanying images. A copy of the written consent is available for review by the Editor-in-Chief of this journal.

## Competing interests

The authors claim no conflict of interest to declare.

## Authors’ contribution

JS-S: performed EBUS and wrote the manuscript. FA: performed EBUS. PS: performed autofluorescence bronchoscopy and attended the patient. ML: performed pathological examination. EC: performed pathological examination. JA: perfomed surgery on the patient. EM: performed EBUS and revised the manuscript. JR-M: revised the manuscript. All authors read and approved the final manuscript.

## Financial support

This paper has been published with funds from a grant from the Spanish Government (FIS 09/01612).
